# Tetra­aqua­bis­[3-(pyridin-4-yl)benzoato-κ*N*]cobalt(II)

**DOI:** 10.1107/S1600536811046496

**Published:** 2011-11-12

**Authors:** Hai-Rong Wang, Guo-Ting Li

**Affiliations:** a Department of Environmental and Municipal Engineering, North China University of Water Conservancy and Electric Power, Zhengzhou 450011, People’s Republic of China

## Abstract

In the title compound, [Co(C_12_H_8_NO_2_)_2_(H_2_O)_4_], the Co atom lies on a twofold rotation axis and has an N_2_O_4_ octa­hedral coordination environment formed by four O atoms of water mol­ecules in the equatorial plane and two apical N atoms of pyridine groups. An intricate three-dimensional supra­molecular network is formed by multiple O—H⋯O hydrogen bonds between the coordinated water mol­ecules and the uncoordinated carboxyl­ate groups.

## Related literature

For the design of metal-organic complexes, see: Ruben *et al.* (2003 ▶). For pyridyl-multicarboxyl­ate-metal frameworks, see: Huang *et al.* (2007 ▶). For similar pyridyl­benzoate complexes, see: Luo *et al.* (2007 ▶). For self-effacement of carboxyl­ate groups in coordination chemistry, see: Lu *et al.* (2008 ▶).
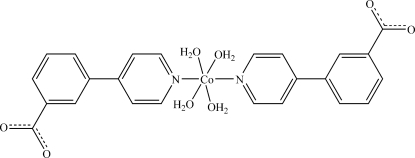


## Experimental

### 

#### Crystal data

[Co(C_12_H_8_NO_2_)_2_(H_2_O)_4_]
*M* *_r_* = 527.38
Monoclinic, 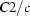

*a* = 24.642 (10) Å
*b* = 7.128 (3) Å
*c* = 13.822 (6) Å
β = 112.660 (7)°
*V* = 2240.4 (16) Å^3^
*Z* = 4
Mo *K*α radiation
μ = 0.82 mm^−1^
*T* = 296 K
0.27 × 0.21 × 0.17 mm

#### Data collection

Siemens SMART CCD diffractometer
4423 measured reflections
1974 independent reflections
1499 reflections with *I* > 2σ(*I*)
*R*_int_ = 0.043

#### Refinement

*R*[*F* ^2^ > 2σ(*F* ^2^)] = 0.049
*wR*(*F* ^2^) = 0.131
*S* = 1.02
1974 reflections
171 parameters
4 restraints
H atoms treated by a mixture of independent and constrained refinement
Δρ_max_ = 0.63 e Å^−3^
Δρ_min_ = −0.43 e Å^−3^

### 

Data collection: *SMART* (Siemens, 1996 ▶); cell refinement: *SAINT* (Siemens, 1994 ▶); data reduction: *SAINT*; program(s) used to solve structure: *SHELXS97* (Sheldrick, 2008 ▶); program(s) used to refine structure: *SHELXL97* (Sheldrick, 2008 ▶); molecular graphics: *SHELXTL* (Sheldrick, 2008 ▶); software used to prepare material for publication: *SHELXL97*.

## Supplementary Material

Crystal structure: contains datablock(s) I, global. DOI: 10.1107/S1600536811046496/yk2023sup1.cif

Structure factors: contains datablock(s) I. DOI: 10.1107/S1600536811046496/yk2023Isup2.hkl

Additional supplementary materials:  crystallographic information; 3D view; checkCIF report

## Figures and Tables

**Table:** 

Co1—O1	2.098 (3)
Co1—O2	2.117 (3)
Co1—N1	2.148 (3)

**Table:** 

O1—Co1—O1^i^	85.18 (15)
O1—Co1—O2	90.11 (11)
O1^i^—Co1—O2	175.22 (10)
O2^i^—Co1—O2	94.61 (15)
O1—Co1—N1	90.43 (10)
O1^i^—Co1—N1	92.54 (10)
O2^i^—Co1—N1	88.98 (10)
O2—Co1—N1	88.28 (10)
O2—Co1—N1^i^	88.98 (10)
N1—Co1—N1^i^	175.97 (15)

Symmetry code: (i) 
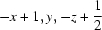
.

**Table 2:** Hydrogen-bond geometry (Å, °)

*D*—H⋯*A*	*D*—H	H⋯*A*	*D*⋯*A*	*D*—H⋯*A*
O1—H1*A*⋯O3^ii^	0.84 (1)	1.89 (2)	2.692 (4)	159 (4)
O2—H2*A*⋯O3^ii^	0.84 (1)	1.94 (2)	2.741 (4)	160 (4)
O1—H1*B*⋯O4^iii^	0.84 (1)	1.91 (1)	2.743 (4)	177 (4)
O2—H2*B*⋯O4^iv^	0.84 (1)	1.89 (1)	2.714 (4)	172 (4)

Symmetry codes: (ii) 
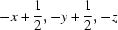
; (iii) 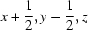; (iv) 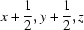.

**Fig. 1.**
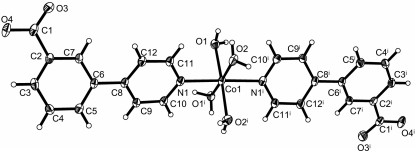
ORTEP diagram of (1) with atom numbering scheme (30% probability ellipsoids for all non-hydrogen atoms). Symmetry code: (i) -x + 1, y, -z + 1/2.

**Fig. 2.**
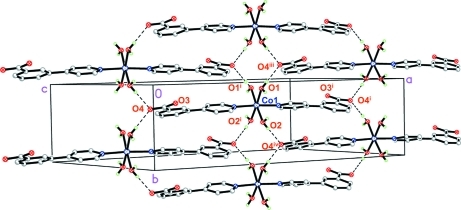
View of regular arrangement of molecules (1) directed by strong hydrogen bonding to form two-dimensional layers and face-to-face π···π stacking interactions between adjacent benzoate and pyridyl groups of PBC. Symmetry codes: (i) -x + 1, y, -z + 1/2; (ii) -x + 1/2, -y + 1/2, -z; (iii) x + 1/2, y - 1/2, z; (iiii) x + 1/2, y + 1/2, z.

## supplementary crystallographic information

### Comment

The synthesis and exploration of multifunctional metal-organic complexes is a current topic of chemical research (M. Ruben, et al., 2003). Pyridyl-containing multi-carboxylic acids, a kind of gracious multifunctional spacers, have been widely used to construct various extended metal-organic frameworks with attracting properties (Y. Huang, et al., 2007). Pyridylbenzoate ligands are typical unsymmetrical spacers, but up to now their coordination chemistry has been discussed uncommonly. From our best knowledge, only one coordination compound of 3-(pyridin-4-yl)benzoic acid (PBC) of pyridylbenzoate family was synthesized and characterized up to now (J. Luo, et al., 2007). Herein we report a new Co(II) complex with PBC, namely, [Co(PBC)_2_(H_2_O)_4_] (1).

As showed in Fig. 1,  (1) is a mononuclear complex with a twofold axis passing through the Co(II) center along b axis and equally splitting the whole complex molecule. In (1), the Co(II) center is ligated by four O atoms of coordinated water molecules in the equatorial plane, whereas two PBC act as monodentate N-donating ligands with their pyridyl nitrogen atoms occupying the axial positions. Thus the cobalt(II) ion is six-coordinated and has octahedral coordination geometry. The bond distances Co—O and Co—N range from 2.098 (3) to 2.148 (3) Å, while the in-plane and axis-transition angles are 175.22 (10) and 175.97 (15) °, respectively, indicating a slight distortion of the octahedral coordination sphere around the Co(II) center.

Owing to the self-effacement of the versatile carboxyl groups in coordination chemistry (W. G.  Lu, et al., 2008), the potential multifunctional  PBC ligands in (1) act as terminal ligand only, rather than bridging one, very dissimilar to that in literature structure (J. Luo, et al., 2007). Further aggregation of monomers (1) is performed by the multiple hydrogen bonds between the coordinated water molecules (as donors) and the uncoordinated carboxylate groups (as acceptors) (Table 1). Hydrogen-bonding system among monomers (1) is rather complicated: each coordination water molecule forms two O—H···O hydrogen bonds with carboxylate groups of neighbouring complex molecules, while every carboxylate group of PBC forms three hydrogen bonds. Consequently, every monomer acts as a novel six-connected supramolecular synthon to connect with six adjacent monomers. For example, as shown in Fig. 2, the O4 of the carboxylate group of PBC ligates to two water molecules from two neighboring monomers, and as a result, monomers (1) are regularly arrayed in the ab<ι> plane and linked into 2D layers by strong hydrogen bonding (O1···O4^iii^, 2.743 (4) Å; O2···O4, 2.714 (4) Å). The layer structure is stabilized by forceful face-to-face π···π stacking interactions between adjacent benzoate and pyridyl groups of PBC with a centroid to centroid distance of 3.60 (1) Å. Intriguingly, the dihedral angle between benzoate and pyridyl groups in PBC is equal to 26.9 (0) ° to meet the formation of hydrogen bonds. The layers are further bound together to create the 3D supramolecular architecture by hydrogen bonds between the O3 atom of the carboxylate group of PBC and two water molecules in the adjacent complex molecule.

### Experimental

The title compound, (1), was prepared according to the following process. A mixture of Co(NO_3_)_2_.6H_2_O (0.029 g, 0.1 mmol), PBC (0.040 g, 0.2 mmol) and deionized water (10 ml) was adjusted to the pH value about 7 by adding 0.1 *M* sodium hydroxide solution and then sealed into a 25 ml Teflon-lined stainless autoclave. The autoclave was heated at 160 °C for 3 days. After cooling to room temperature gradually, dark-red block crystals being characterized as the previously reported complex (J. Luo, *et al.*, 2007) were obtained in 30% yield (based on Co). Allowing the red filtrate to evaporate slowly at ambient temperature for two months, pink crystals of (1) suitable for X-ray analysis were obtained in 58% yield (based on Co).

### Refinement

The H atoms of water were located from the difference Fourier maps and included in the final refinement by using geometrical restrains, while the other hydrogen atom positions were generated geometrically and these H atoms were allowed to ride on their parent atoms.

### Crystal data

**Table:**

[Co(C_12_H_8_NO_2_)_2_(H_2_O)_4_]	*F*(000) = 1092
*M**_r_* = 527.38	*D*_x_ = 1.564 Mg m^−^^3^
Monoclinic, *C*2/*c*	Mo *K*α radiation, λ = 0.71073 Å
Hall symbol: -C 2yc	Cell parameters from 981 reflections
*a* = 24.642 (10) Å	θ = 3.0–21.3°
*b* = 7.128 (3) Å	µ = 0.82 mm^−^^1^
*c* = 13.822 (6) Å	*T* = 296 K
β = 112.660 (7)°	Block, red
*V* = 2240.4 (16) Å^3^	0.27 × 0.21 × 0.17 mm
*Z* = 4	

### Data collection

**Table:**

Siemens SMART CCD diffractometer	1499 reflections with *I* > 2σ(*I*)
Radiation source: fine-focus sealed tube	*R*_int_ = 0.043
graphite	θ_max_ = 25.0°, θ_min_ = 1.8°
ω scan	*h* = −23→29
4423 measured reflections	*k* = −8→8
1974 independent reflections	*l* = −14→16

### Refinement

**Table:**

Refinement on *F*^2^	Primary atom site location: structure-invariant direct methods
Least-squares matrix: full	Secondary atom site location: difference Fourier map
*R*[*F*^2^ > 2σ(*F*^2^)] = 0.049	Hydrogen site location: inferred from neighbouring sites
*wR*(*F*^2^) = 0.131	H atoms treated by a mixture of independent and constrained refinement
*S* = 1.02	*w* = 1/[σ^2^(*F*_o_^2^) + (0.0755*P*)^2^] where *P* = (*F*_o_^2^ + 2*F*_c_^2^)/3
1974 reflections	(Δ/σ)_max_ < 0.001
171 parameters	Δρ_max_ = 0.63 e Å^−^^3^
4 restraints	Δρ_min_ = −0.43 e Å^−^^3^

### Special details

**Table:**

Geometry. All e.s.d.'s (except the e.s.d. in the dihedral angle between two l.s. planes) are estimated using the full covariance matrix. The cell e.s.d.'s are taken into account individually in the estimation of e.s.d.'s in distances, angles and torsion angles; correlations between e.s.d.'s in cell parameters are only used when they are defined by crystal symmetry. An approximate (isotropic) treatment of cell e.s.d.'s is used for estimating e.s.d.'s involving l.s. planes.
Refinement. Refinement of *F*^2^ against ALL reflections. The weighted *R*-factor *wR* and goodness of fit *S* are based on *F*^2^, conventional *R*-factors *R* are based on *F*, with *F* set to zero for negative *F*^2^. The threshold expression of *F*^2^ > σ(*F*^2^) is used only for calculating *R*-factors(gt) *etc*. and is not relevant to the choice of reflections for refinement. *R*-factors based on *F*^2^ are statistically about twice as large as those based on *F*, and *R*-factors based on ALL data will be even larger.

### Fractional atomic coordinates and isotropic or equivalent isotropic displacement parameters (Å^2^)

**Table:**

	*x*	*y*	*z*	*U*_iso_*/*U*_eq_	
Co1	0.5000	0.30197 (9)	0.2500	0.0294 (3)	
O1	0.46997 (11)	0.0853 (4)	0.13933 (19)	0.0381 (6)	
O2	0.46824 (11)	0.5033 (4)	0.1285 (2)	0.0407 (6)	
O3	0.09455 (11)	0.2256 (4)	0.0359 (2)	0.0515 (8)	
O4	0.03868 (11)	0.2931 (4)	0.1240 (2)	0.0455 (7)	
N1	0.41603 (12)	0.3126 (4)	0.2633 (2)	0.0322 (7)	
C1	0.08769 (15)	0.2741 (5)	0.1173 (3)	0.0355 (9)	
C2	0.14247 (15)	0.3156 (5)	0.2136 (3)	0.0306 (8)	
C3	0.13890 (16)	0.3628 (5)	0.3077 (3)	0.0383 (9)	
H3	0.1017	0.3689	0.3134	0.046*	
C4	0.18993 (16)	0.4014 (6)	0.3940 (3)	0.0404 (9)	
H4	0.1876	0.4354	0.4587	0.049*	
C5	0.24431 (15)	0.3905 (5)	0.3862 (3)	0.0365 (8)	
H5	0.2790	0.4165	0.4459	0.044*	
C6	0.24867 (14)	0.3419 (5)	0.2920 (3)	0.0297 (8)	
C7	0.19680 (14)	0.3043 (5)	0.2059 (3)	0.0302 (8)	
H7	0.1988	0.2703	0.1409	0.036*	
C8	0.30666 (14)	0.3304 (5)	0.2826 (3)	0.0300 (8)	
C9	0.35800 (15)	0.2917 (5)	0.3676 (3)	0.0342 (8)	
H9	0.3569	0.2698	0.4346	0.041*	
C10	0.41091 (15)	0.2848 (5)	0.3551 (3)	0.0347 (8)	
H10	0.4456	0.2590	0.4149	0.042*	
C11	0.36609 (15)	0.3493 (5)	0.1809 (3)	0.0355 (9)	
H11	0.3683	0.3688	0.1144	0.043*	
C12	0.31186 (15)	0.3603 (5)	0.1876 (3)	0.0364 (9)	
H12	0.2779	0.3884	0.1269	0.044*	
H1A	0.4545 (17)	0.127 (6)	0.0779 (15)	0.055*	
H1B	0.4919 (15)	−0.001 (4)	0.136 (3)	0.055*	
H2B	0.4916 (15)	0.591 (4)	0.133 (3)	0.055*	
H2A	0.4530 (17)	0.448 (6)	0.0706 (18)	0.055*	

### Atomic displacement parameters (Å^2^)

**Table:**

	*U*^11^	*U*^22^	*U*^33^	*U*^12^	*U*^13^	*U*^23^
Co1	0.0181 (4)	0.0332 (4)	0.0371 (4)	0.000	0.0107 (3)	0.000
O1	0.0348 (15)	0.0390 (16)	0.0428 (14)	0.0036 (12)	0.0175 (12)	−0.0014 (12)
O2	0.0293 (14)	0.0402 (17)	0.0484 (15)	−0.0077 (11)	0.0101 (12)	0.0059 (13)
O3	0.0313 (15)	0.074 (2)	0.0434 (15)	0.0003 (13)	0.0077 (12)	−0.0062 (14)
O4	0.0228 (14)	0.0398 (15)	0.0731 (19)	0.0005 (11)	0.0173 (13)	−0.0009 (13)
N1	0.0222 (15)	0.0333 (17)	0.0402 (17)	0.0000 (12)	0.0110 (13)	−0.0020 (13)
C1	0.0241 (19)	0.0287 (19)	0.051 (2)	0.0019 (15)	0.0120 (16)	0.0076 (17)
C2	0.0240 (18)	0.0275 (18)	0.0398 (18)	0.0035 (14)	0.0118 (15)	0.0058 (15)
C3	0.031 (2)	0.043 (2)	0.048 (2)	0.0082 (16)	0.0229 (17)	0.0083 (17)
C4	0.034 (2)	0.054 (3)	0.0377 (19)	0.0040 (18)	0.0179 (16)	−0.0032 (18)
C5	0.0264 (19)	0.043 (2)	0.039 (2)	−0.0014 (16)	0.0107 (15)	−0.0029 (17)
C6	0.0219 (17)	0.0283 (19)	0.0401 (19)	0.0009 (14)	0.0133 (15)	0.0010 (15)
C7	0.0247 (18)	0.0315 (19)	0.0367 (18)	−0.0017 (15)	0.0146 (15)	−0.0006 (15)
C8	0.0210 (17)	0.0280 (19)	0.0416 (19)	−0.0007 (14)	0.0128 (15)	−0.0026 (15)
C9	0.0242 (18)	0.047 (2)	0.0324 (18)	−0.0023 (16)	0.0114 (15)	−0.0004 (16)
C10	0.0222 (18)	0.043 (2)	0.0361 (19)	−0.0020 (15)	0.0081 (15)	−0.0005 (16)
C11	0.0224 (18)	0.048 (2)	0.0370 (19)	−0.0006 (15)	0.0122 (15)	0.0002 (16)
C12	0.0238 (18)	0.044 (2)	0.040 (2)	0.0017 (16)	0.0114 (15)	−0.0008 (17)

### Geometric parameters (Å, °)

**Table:**

Co1—O1	2.098 (3)	C3—C4	1.387 (5)
Co1—O1^i^	2.098 (3)	C3—H3	0.9500
Co1—O2^i^	2.117 (3)	C4—C5	1.387 (5)
Co1—O2	2.117 (3)	C4—H4	0.9500
Co1—N1	2.148 (3)	C5—C6	1.390 (5)
Co1—N1^i^	2.148 (3)	C5—H5	0.9500
O1—H1A	0.840 (10)	C6—C7	1.396 (5)
O1—H1B	0.836 (10)	C6—C8	1.487 (5)
O2—H2B	0.835 (10)	C7—H7	0.9500
O2—H2A	0.840 (10)	C8—C9	1.383 (5)
O3—C1	1.249 (5)	C8—C12	1.383 (5)
O4—C1	1.254 (4)	C9—C10	1.381 (5)
N1—C10	1.337 (4)	C9—H9	0.9500
N1—C11	1.342 (4)	C10—H10	0.9500
C1—C2	1.516 (5)	C11—C12	1.377 (5)
C2—C3	1.378 (5)	C11—H11	0.9500
C2—C7	1.386 (5)	C12—H12	0.9500
			
O1—Co1—O1^i^	85.18 (15)	C2—C3—C4	119.6 (3)
O1—Co1—O2^i^	175.22 (10)	C2—C3—H3	120.2
O1^i^—Co1—O2^i^	90.11 (11)	C4—C3—H3	120.2
O1—Co1—O2	90.11 (11)	C3—C4—C5	120.3 (3)
O1^i^—Co1—O2	175.22 (10)	C3—C4—H4	119.8
O2^i^—Co1—O2	94.61 (15)	C5—C4—H4	119.8
O1—Co1—N1	90.43 (10)	C4—C5—C6	120.8 (3)
O1^i^—Co1—N1	92.54 (10)	C4—C5—H5	119.6
O2^i^—Co1—N1	88.98 (10)	C6—C5—H5	119.6
O2—Co1—N1	88.28 (10)	C5—C6—C7	118.0 (3)
O1—Co1—N1^i^	92.54 (10)	C5—C6—C8	121.3 (3)
O1^i^—Co1—N1^i^	90.43 (10)	C7—C6—C8	120.6 (3)
O2^i^—Co1—N1^i^	88.28 (10)	C2—C7—C6	121.2 (3)
O2—Co1—N1^i^	88.98 (10)	C2—C7—H7	119.4
N1—Co1—N1^i^	175.97 (15)	C6—C7—H7	119.4
Co1—O1—H1A	112 (3)	C9—C8—C12	116.6 (3)
Co1—O1—H1B	122 (3)	C9—C8—C6	122.1 (3)
H1A—O1—H1B	104 (4)	C12—C8—C6	121.2 (3)
Co1—O2—H2B	114 (3)	C10—C9—C8	119.9 (3)
Co1—O2—H2A	109 (3)	C10—C9—H9	120.0
H2B—O2—H2A	118 (4)	C8—C9—H9	120.0
C10—N1—C11	116.3 (3)	N1—C10—C9	123.5 (3)
C10—N1—Co1	121.5 (2)	N1—C10—H10	118.2
C11—N1—Co1	122.2 (2)	C9—C10—H10	118.2
O3—C1—O4	124.4 (4)	N1—C11—C12	123.3 (3)
O3—C1—C2	117.5 (3)	N1—C11—H11	118.3
O4—C1—C2	118.1 (3)	C12—C11—H11	118.3
C3—C2—C7	120.1 (3)	C11—C12—C8	120.2 (3)
C3—C2—C1	121.1 (3)	C11—C12—H12	119.9
C7—C2—C1	118.8 (3)	C8—C12—H12	119.9

Symmetry codes: (i) −*x*+1, *y*, −*z*+1/2.

### Hydrogen-bond geometry (Å, °)

**Table:**

*D*—H···*A*	*D*—H	H···*A*	*D*···*A*	*D*—H···*A*
O1—H1A···O3^ii^	0.84 (1)	1.89 (2)	2.692 (4)	159 (4)
O2—H2A···O3^ii^	0.84 (1)	1.94 (2)	2.741 (4)	160 (4)
O1—H1B···O4^iii^	0.84 (1)	1.91 (1)	2.743 (4)	177 (4)
O2—H2B···O4^iv^	0.84 (1)	1.89 (1)	2.714 (4)	172 (4)

Symmetry codes: (ii) −*x*+1/2, −*y*+1/2, −*z*; (iii) *x*+1/2, *y*−1/2, *z*; (iv) *x*+1/2, *y*+1/2, *z*.

## References

[bb1] Huang, Y., Wu, B., Yuan, D., Xu, Y., Jiang, F. & Hong, M. (2007). *Inorg. Chem.* **46**, 1171–1176. 10.1021/ic0615271 17291113

[bb2] Lu, W. G., Jiang, L., Feng, X. L. & Lu, T. B. (2008). *Cryst. Growth Des.* **8**, 986–994.

[bb3] Luo, J., Zhao, Y., Xu, H., Kinibrugh, T. L., Yang, D., Timofeeva, T. V., Daemen, L. L., Zhang, J., Bao, W., Thompson, J. D. & Currier, R. P. (2007). *Inorg. Chem.* **46**, 9021–9023. 10.1021/ic701003z 17887748

[bb4] Ruben, M., Breuning, E., Barboiu, M., Gisselbrecht, J.-P. & Lehn, J.-M. (2003). *Chem. Eur. J.* **9**, 291–299. 10.1002/chem.200390022 12506385

[bb5] Sheldrick, G. M. (2008). *Acta Cryst.* A**64**, 112–122. 10.1107/S0108767307043930 18156677

[bb6] Siemens (1994). *SAINT* Siemens Analytical X-ray Instruments Inc., Madison, Wisconsin, USA.

[bb7] Siemens (1996). *SMART* Siemens Analytical X-ray Instruments Inc., Madison, Wisconsin, USA.

